# Combining Polygenic Risk Score and Voice Features to Detect Major Depressive Disorders

**DOI:** 10.3389/fgene.2021.761141

**Published:** 2021-12-20

**Authors:** Yazheng Di, Jingying Wang, Xiaoqian Liu, Tingshao Zhu

**Affiliations:** ^1^ Key Laboratory of Behavioral Science, Institute of Psychology, Chinese Academy of Sciences, Beijing, China; ^2^ Department of Psychology, University of Chinese Academy of Sciences, Beijing, China; ^3^ School of Optometry, Faculty of Health and Social Sciences, Hong Kong Polytechnic University, Hong Kong, China

**Keywords:** biomarkers, polygenic risk score (PRS), computer technology, major depressive disorder (MDD), voice biomarkers, depression

## Abstract

**Background:** The application of polygenic risk scores (PRSs) in major depressive disorder (MDD) detection is constrained by its simplicity and uncertainty. One promising way to further extend its usability is fusion with other biomarkers. This study constructed an MDD biomarker by combining the PRS and voice features and evaluated their ability based on large clinical samples.

**Methods:** We collected genome-wide sequences and utterances edited from clinical interview speech records from 3,580 women with recurrent MDD and 4,016 healthy people. Then, we constructed PRS as a gene biomarker by *p* value-based clumping and thresholding and extracted voice features using the i-vector method. Using logistic regression, we compared the ability of gene or voice biomarkers with the ability of both in combination for MDD detection. We also tested more machine learning models to further improve the detection capability.

**Results:** With a *p*-value threshold of 0.005, the combined biomarker improved the area under the receiver operating characteristic curve (AUC) by 9.09% compared to that of genes only and 6.73% compared to that of voice only. Multilayer perceptron can further heighten the AUC by 3.6% compared to logistic regression, while support vector machine and random forests showed no better performance.

**Conclusion:** The addition of voice biomarkers to genes can effectively improve the ability to detect MDD. The combination of PRS and voice biomarkers in MDD detection is feasible. This study provides a foundation for exploring the clinical application of genetic and voice biomarkers in the diagnosis of MDD.

## 1 Introduction

The deployment of bioinformatic evaluations in psychiatry would revolutionize the ability to diagnose, treat, and prevent major depressive disorder (MDD). MDD affects nearly 1 in 10 people (Kessler et al., 2003; Demyttenaere et al., 2004) and has lately been recognized as the world’s leading cause of disability (World Health Organization, 2017). However, only approximately half of the population suffering from MDD is currently identified and treated (Wells et al., 1989; Goldberg 1995). The difficulty in identifying MDD is one of the key barriers to the effective utilization of current medications. Diagnosis remains based on clinical interviews and mental status examination (Regier et al., 2013); screening instruments are hindered by poor specificity and sensitivity, and there are no reliable biomarkers. Furthermore, because MDD is a syndromic diagnosis, it possibly comprises several different diseases, each with its own set of symptoms and treatment response (Alexopoulos et al., 1997; Kendler et al., 2001, 2006; Kendler et al., 2013; Gustafsson et al., 2015; Masters et al., 2015; Peterson et al., 2018).

The study of constructing MDD biomarkers has shown two different orientations. On the one hand, researchers have been devoted to finding the biological basis of depression (Schneider and Prvulovic 2013), for example, genetic factors (23andMe Research Team et al., 2019; CONVERGE Consortium, 2015; eQTLGen et al., 2018), on which to build valid biomarkers. On the other hand, studies have started from behavioral indices that are easily accessible and nonintrusive, such as patient speech voice (Low et al., 2020). The studies focus on improving diagnostic accuracy by developing machine learning (ML) algorithms.

Researchers have spent decades looking for the genetic foundation for developing more accurate MDD diagnosis models (Reus et al., 2017; Mullins et al., 2019; Rantalainen et al., 2020). The results from genome-wide association studies (GWAS) (23andMe Research Team et al., 2019; CONVERGE Consortium, 2015; eQTLGen et al., 2018) suggested that MDD is polygenic, which means that hundreds of DNA variants impact its hereditary influences with very small effects. Polygenic risk scores (PRSs) provide an estimated risk for individuals suffering from MDD. PRS is calculated as a weighted sum of an individual’s risk alleles, where their weights are specified by loci and their assessed effects found by GWAS (Chatterjee et al., 2016). Advances in biotechnology have made sequencing technologies less expensive and the genetic screening of individuals easier. However, the utility of PRS in MDD prediction is currently constrained by its simplicity and uncertainty, which, to date, captures only part of the genetic contribution to MDD risk (Murray et al., 2021). Moreover, other non-genetic risk factors, such as lifestyles, also play important roles in MDD. As a result, extending the PRS models with other MDD biomarkers may be a more practical solution to addressing this problem (Torkamani et al., 2018).

Benefitting from the development of speech recognition technology, voice-based diagnostic models for depression have been validated and have achieved a high level of accuracy. Speech biomarkers can be used not only to identify depression (Low et al., 2020) but also to recognize the severity of depression (Shin et al., 2021) and predict depression-related symptoms (Zhang et al., 2020). One of the main obstacles hindering the application of voice biomarkers is its poor generalization ability, as traditional voice feature distribution can easily change due to different speech content and speakers (Wang et al., 2019). To address this issue, researchers developed the i-vector method, extracting the factors from voice features that are independent of speaker and channel variabilities (Dehak et al., 2011; Cummins et al., 2014). A study recognizing MDD in 1,808 clinical samples proved that voice i-vectors are effective and robust (Di et al., 2021). Therefore, combining technologies in speech recognition and integrating them into existing genetic models are likely to enable clinical diagnosis in general populations.

To construct biomarkers for clinical disease detection, researchers have combined PRS with known risk factors (Hoang et al., 2021; Kapoor et al., 2021), neuroimaging, metabolites (Badhwar et al., 2020), or body indicators (Moldovan et al., 2021). However, to the best of our knowledge, there are no studies combining genes and voice in detecting MDD, which may be due to the difficulty in obtaining multiple types of samples of the same subject simultaneously. Evidence from clinical samples is needed to prove their ecological validity (Zhang et al., 2020; Murray et al., 2021). The combination and cross-validation of biological and behavioral biomarkers hold great promise to take us one step closer to the objective clinical diagnosis of MDD.

Here, based on a large sample of women with recurrent MDD diagnosed clinically, we used the PRS together with voice i-vectors to detect MDD. We examined whether their combination could surpass a single biomarker. We constructed models on different single nucleotide polymorphisms (SNPs) to examine their robustness. We also tested various ML models to find the better model.

## 2 Materials and Methods

**FIGURE 1:**
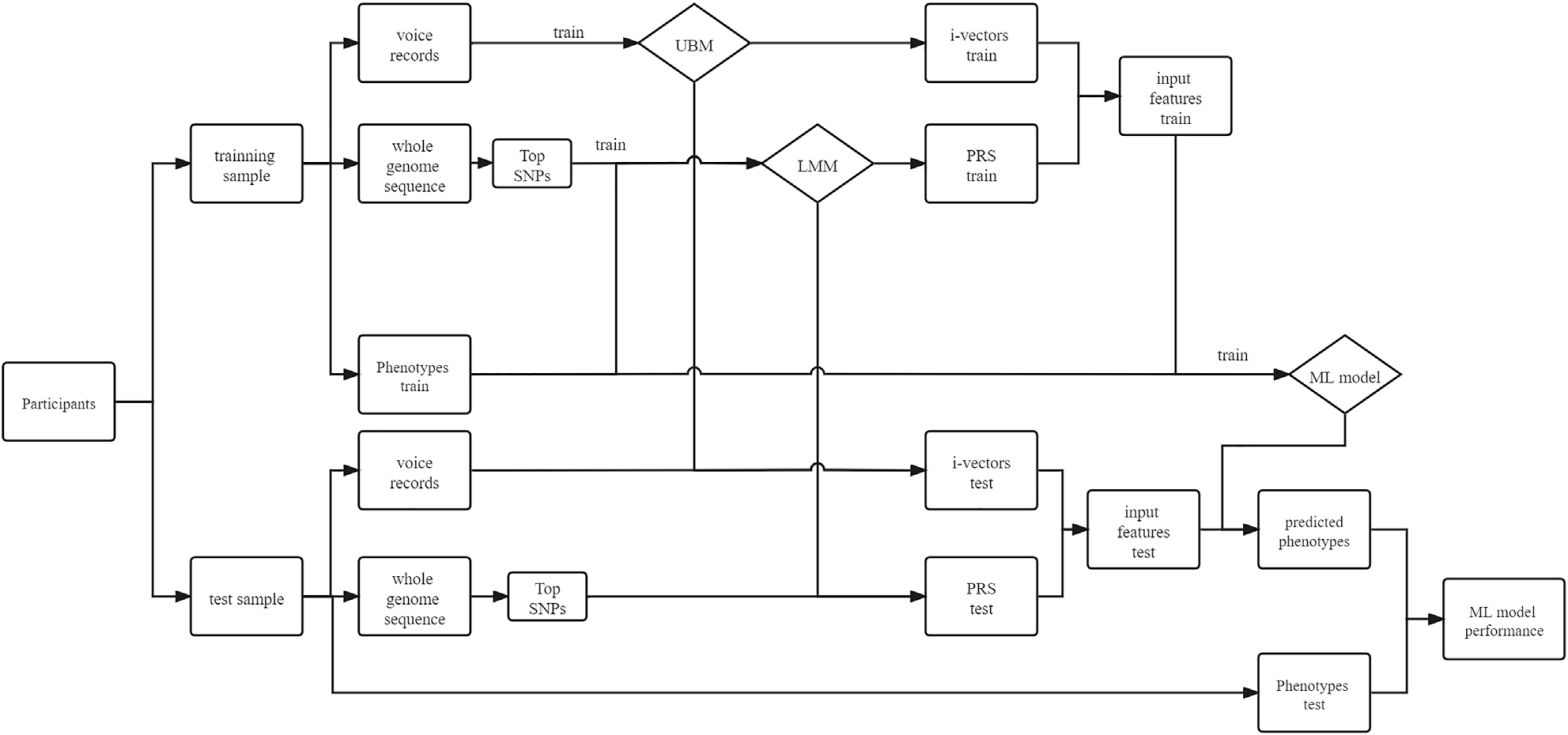
Fivefold cross-validation of voice–gene data. In each fold, the samples were split into a training group and a test group. Voice and genetic sequence data of the training group were used to train the universal background model (UBM) and linear mixed model (LMM) separately. Then, i-vectors for the training and test groups were extracted through the UBM, and the polygenic risk score (PRS) can be calculated through the LMM. The i-vectors and PRS will be concatenated as input features for a machine learning (ML) model.

We used a fivefold cross-validation design in this study. As shown in Figure 1, we split 80% of the samples into a training group and the rest into a test group. Firstly, we used voice data from the training samples to train the universal background model (UBM), and we used this UBM to extract i-vectors for each individual. Then, we used clumped SNP data from the training samples to train the PRS model, through which we calculated the PRS for each individual. Finally, we used the PRS and voice i-vectors from the training samples to train the ML models and used the same features from the test samples to validate the model performance. The details of each step are explained below.

### 2.1 Data Collection

The database used in this study was developed from the China, Oxford, and Virginia Commonwealth University Experimental Research on Genetic Epidemiology (CONVERGE). The CONVERGE study, designed for a genome-wide association of major depression disorders, recruited 11,670 Han Chinese women. There were 5,303 women with recurrent MDD aged between 30 and 60 years whose first episodes of MDD met the DSM-IV criteria (Association 1994). A total of 5,337 controls were recruited from patients undergoing minor surgical procedures at general hospitals or from local community centers. Only women were included in this study to minimize genetic heterogeneity because approximately 45% of the genetic liability to MDD is not shared between sexes (Kendler et al., 2007; Sullivan et al., 2000). The subject inclusion criteria and interview process were strictly controlled, as detailed in CONVERGE Consortium (2015).

The voice data of the patients were from the records during the semi-structured interview, which included assessments of psychopathology, demographic and personal characteristics, and psychosocial functioning. These voice data are characterized by a high degree of phonetic and content variety. A detailed description of the interview protocol is in Di et al. (2021).

### 2.2 Data Preprocessing

#### 2.2.1 Genetic Data

DNA sequencing, variant calling, and the genotype likelihood calculation and imputation processes are described in CONVERGE Consortium, (2015). We used PLINK (Chang et al., 2015) to select SNPs with minor allele frequency (MAF) >0.5% and imputation quality INFO score >0.9 and clumped the SNP set using *r* ^2^ = 0.5 with 50-kb windows. A total of 359,515 SNPs passed the filter.

#### 2.2.2 Voice Data

The utterances of participants were edited from recordings of the conversations between doctors and patients through the following steps. Firstly, voice segments from the participants were selected and labeled. Then, all the segments of one participant were combined into one utterance. Due to the variety of interviews, not all voice samples of participants had segments >2 s for the latter analysis. Thus, samples with both genetic data and enough voice data were passed to subsequent analysis, and the total number was 7,596 (3,580 cases and 4,016 controls). All utterances were downsampled to 8 kHz for subsequent processing.

### 2.3 Data Analysis

#### 2.3.1 PRS Models

We used the linear mixed model (Li and Zhu 2013) to calculate the PRS. The model can be written as follows:

y=Xβ+g+e

Here, X is the matrix of the fixed effects, including covariates and the genetic matrix; the vector β is the coefficient of fixed effects; g is a random effect reflecting polygene background; and e denotes the random residual effect.

We used the *p* value-based thresholding (P+T) method (Wray et al, 2007) to construct the PRS model. Usually, a lower threshold than genome-wide statistical significance can be applied to increase the overall predictability, generally at the sacrifice of generalizability (Murray et al., 2021). Different *p*-value thresholds (PTs) were tested, ranging from 5 × 10^−8^ to 5 × 10^−3^ (10^−3^ is a conservative significance threshold of *p* suggested by Euesden et al., 2015). The PRS model was trained using the FaST-LMM (Lippert et al., 2011) predictor, which efficiently reduced the computational time.

To assess how the confounders affect the model’s predictability and generalizability, we considered the following covariates: age, education, occupation, social class, marital status, height, weight, and 40 genetic principal components. We compared three different covariate use strategies. The first was a model ignoring the covariates (no-cov), the second was trained by and predicted on the genetic matrix along with covariates (all-cov), and the last was a model trained by a genetic matrix along with true covariates of the training samples, but made predictions on test samples whose covariate values were replaced with random numbers (random-cov).

#### 2.3.2 Voice i-Vectors

**FIGURE 2:**
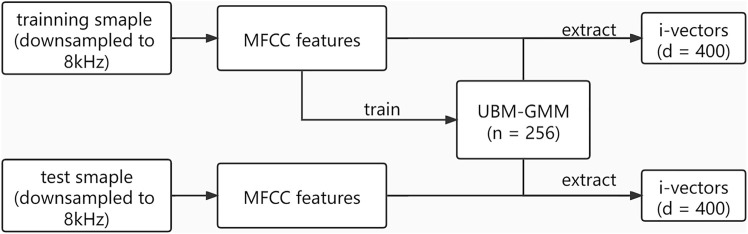
Process of i-vector extraction. UBM-GMM is a universal background model adapted by a Gaussian mixture model. *n* = 256 means there were 256 Gaussian mixture clusters. *d* = 400 means the dimension of i-vectors is 400.

The i-vector extraction process is shown in Figure 2. Firstly, mel frequency cepstral coefficients (MFCCs) were extracted with a window size of 25 ms, a window shift of 10 ms, a pre-emphasis filter with a coefficient of 0.97, and a sinusoidal lifter with a coefficient of 22. A filter bank with 23 filters was used, and 12 coefficients were extracted. Then, for the given voice features, we set the number of Gaussian mixtures as 256 to estimate the utterance-dependent Gaussian mixture model (GMM) parameters and adapted the UBM (Kenny et al., 2005), which represents the feature distribution of the acoustic space.

The i-vectors are low-dimensional representations of the voice features based on factor analysis (Kenny et al., 2008), onto which the acoustic space is mapped via a linear transformation while keeping the majority of the variability inherent in the acoustic space. This approach has been widely used in speaker verification. The i-vector method (Dehak et al., 2011) can be expressed as follows:

M=m+Tv

where ***m*** is the mean supervector of the UBM. For the purpose of depression classification, it is expected that the UBM approximately models the phonetic variability of the acoustic space. ***M*** is the mean centered supervector of the speech utterance derived using the zeroth- and first-order Baum–Welch statistics. ***v*** is the i-vector, which captures variations in this structure caused by other factors, such as depression level, speaker identity, and channel effects (Cummins et al., 2014). We used the Kaldi speech recognition toolkit (Povey et al., 2011) and extracted the 400 dimensions of i-vectors.

#### 2.3.3 ML Models

We used a logistic regression (LR) classifier as a benchmark model, for which PRS, i-vectors, and both were used as input features. Then, we used random forest (RF), support vector machine (SVM), and multilayer perceptron (MLP) classifiers to test whether there was an improvement compared to the benchmark. We report the sensitivity, specificity, and area under the receiver operator characteristic curve (AUC) from the fivefold cross-validation. We used scikit-learn (Pedregosa et al., 2011) for the above process.

For the LR model, we also divided the test samples into the top 25%, middle 50%, and bottom 25% according to their PRS and calculated the accuracy on each stratification to test whether the accuracy of the biomarkers remains consistent across different genetic risk stratifications.

#### 2.3.4 Binary Logistic Regression

To check the contribution of voice and genes separately, we built three logistic regression models using a conditional forward step. Taking MDD as the dependent variable, voice i-vectors, PRS, and the combination of both were entered into the model separately as independent variables. Nagelkerke’s *R* ^2^ (Nagelkerke, 1991) was utilized as an indicator of the contributing effect of the variables.

## 3 Results

### 3.1 PRS Model and Covariates

**FIGURE 3:**
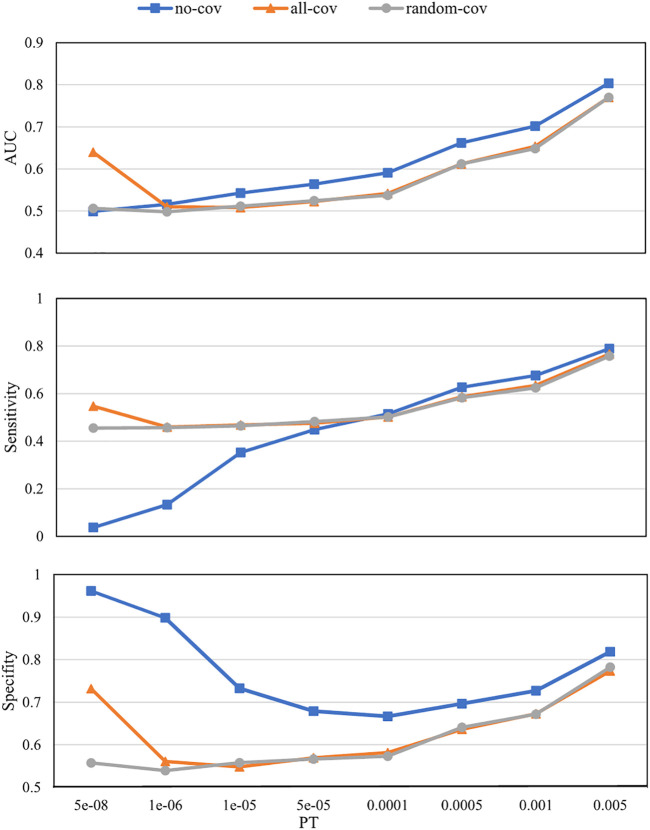
Polygenic risk score (PRS) model prediction results with different *p*-value thresholds (PTs) under different covariate use strategies. *no-cov*, no covariates were considered during the training and prediction processes; *all-cov*, all covariates were considered during the training and prediction processes; *random-cov*, the PRS model was trained with a sample genetic matrix along with covariates, but made predictions on samples whose covariates were replaced with random numbers. AUC, Area under the receiver operating characteristic curve.

The numbers of SNPs selected using different PTs are shown in Table 1. The SNPs and their estimated weights in previous GWAS (CONVERGE Consortium 2015) are provided in Supplementary Data Sheet S1. Figure 3 shows the detection ability of the PRS models with different PTs using different covariate use strategies. When $\mathrm{PT}=5\times 10^{-8}$ , PRS with all-cov achieved the best AUC (0.64), while the other two were close to random guessing (0.50). When $\mathrm{PT>}5\times 10^{-8}$ , PRS with no-cov consistently achieved better AUC than did PRS with all-cov and random-cov, and the performances of PRS with random-cov and all-cov were close.

**TABLE 1:** Number of SNPs selected on different *p*-value thresholds (PTs)

PT	5E−08	1E−06	1E−5	5E−5	1E−4	5E−4	1E−3	5E−3
*N*	3	5	11	44	79	321	580	2,350

### 3.2 Prediction Results Using Different Biomarkers

**FIGURE 4:**
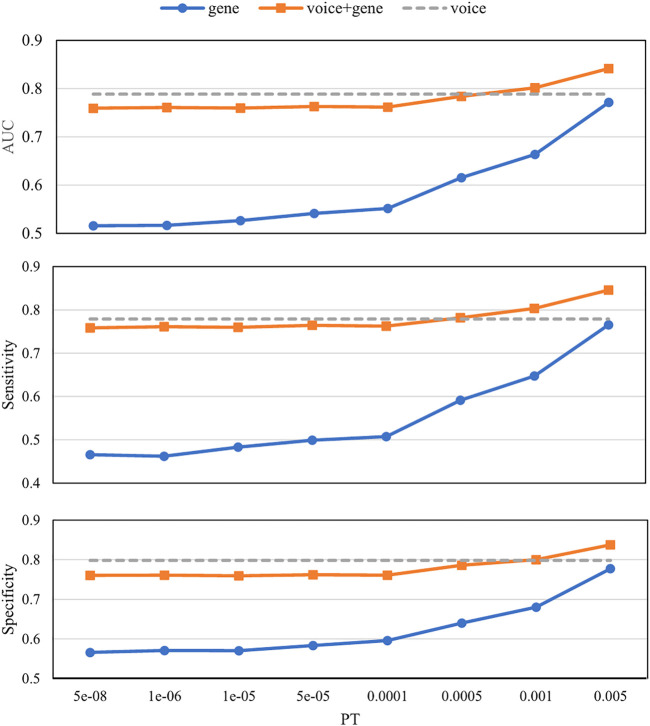
Prediction results with different *p*-value thresholds (PTs) using different biomarkers. The *x*-axis is the *p*-value threshold (PT) used in the gene model and the combined biomarkers. Voice biomarkers are not related to PT and are indicated by a *dashed horizontal line*. AUC, Area under the receiver operating characteristic curve.

Figure 4 shows the prediction results with different PTs using different biomarkers. The voice biomarkers achieved an AUC of 0.79. With the decrease in PT, the AUCs for genes only and the combined biomarkers both increased. Compared with genes only, the combined biomarkers always performed better. Compared with voice only, the combined biomarkers did not win until PT > 0.0005.

### 3.3 Binary Logistic Regression

We examined how much gene and voice contributed to MDD using Nagelkerke’s *R* ^2^. For voice only and gene only, Nagelkerke’s *R* ^2^ values were 0.571 and 0.829, respectively. For the combined biomarkers, Nagelkerke’s *R* ^2^ was 0.902. Details of the logistic regression models are in Supplementary Data Sheet S2.

**FIGURE 5:**
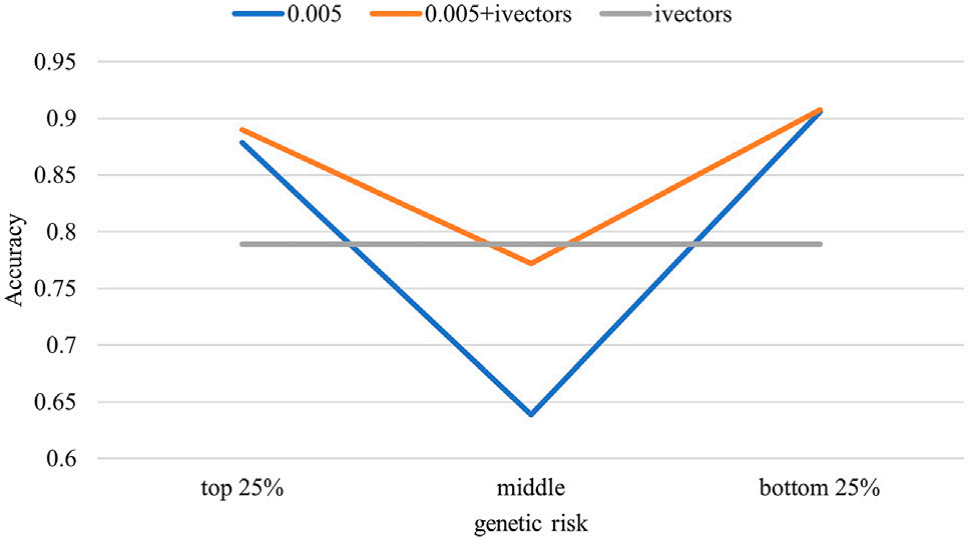
Stratified population accuracy using different biomarkers. The test samples were divided into three groups according to their predicted polygenic risk scores (PRSs). Accuracies were calculated for the three groups separately.

Figure 5 shows the stratified accuracies of the different biomarkers in predicting MDD. The voice biomarker performed consistently in the three stratifications with different genetic risks, all at 0.79. However, the accuracy of genes varied considerably between the middle (0.64) and the two ends of the population (close to 0.9). The combined biomarker performed as well as the genes in the two ends and as well as the voice in the middle.

### 3.4 Classification Results Using Different ML Models

The classification results using LR, SVM, RF, and MLP are shown in Table 2. Two PTs (0.001 and 0.005) are presented here, while the results with more PTs are shown in Supplementary Data Sheet S3. The AUCs of LR were 0.79 and 0.83 at the two PTs. Taking LR as a benchmark, MLP achieved better results, with AUCs of 0.81 and 0.86 at the two PTs. The performance of SVM was close to that of LR, and that of RF was worse than that of LR.

**TABLE 2:** Classification results using different machine learning (ML) models

	Gene (PT = 0.001) + voice			Gene (PT = 0.005) + voice		
	AUC	Sensitivity	Specificity	AUC	Sensitivity	Specificity
LR	0.79	0.79	0.78	0.83	0.83	0.83
SVM	0.79	0.80	0.78	0.83	0.83	0.83
RF	0.74	0.70	0.77	0.80	0.78	0.81
MLP	0.81	0.83	0.79	0.86	0.87	0.85

PT, p-value threshold; AUC, area under the receiver operating characteristic curve; LR, logistic regression; SVM, support vector machine; RF, random forest; MLP, multilayer perceptron

## 4 Discussion

This study combines the PRS and voice i-vectors to evaluate their ability to detect MDD. PRSs were calculated at different PTs. Using logistic regression, we compared the abilities of single biomarkers with the combined biomarker for MDD detection. We stratified the test group by genetic risk and examined whether the detection ability differed between stratifications. We also tested various ML models to find the best model.

A good PRS model would have high predictability, contributed mainly by capturing causal genetic variants instead of confounds. The estimated genetic fixed effect may be erroneously high for a linear mixed model if the confounding effects are not estimated. Thus, similar to our data from the same cohort, the no-cov PRS model always performed better than the all-cov PRS model (Figure 3). We believe that the results from the no-cov model are not capable of reflecting real situations because, in practical clinical applications, the distribution of covariates for a newly arrived patient is likely to be different from the distribution of the patients in our training set.

A comparison of PRS with the all-cov and random-cov models can demonstrate how the covariates affect the final prediction results in this study. When $\mathrm{PT=}5\times 10^{-8}$ , the all-cov PRS achieved an AUC of 0.65, while the other two were close to random guessing, which indicated that the covariates were the main contributors to the predictor when there were few SNPs. When $\mathrm{PT>}5\times 10^{-8}$ , the all-cov PRS showed ability equivalent to that of the random-cov PRS, which suggested that the covariates contributed very little to the predictor when the SNP number increased. The covariate analysis suggests two conclusions. Firstly, we must consider the covariates in the training process; otherwise, the performance will be erroneously better than that in actual situations. Secondly, in practical clinical applications, covariate information is not necessary.

The prediction results using different biomarkers demonstrated the ability of these biomarkers to detect MDD (Figure 4). The AUC of voice biomarkers was 0.79, which is consistent with our previous study on 1,808 clinical samples (Di et al., 2021). Since our previous study investigated the meaning of voice i-vectors, in this study, we attended to comparing its performance with the combination of PRS. Compared with genes only, the combined biomarkers can significantly improve the predictive ability at all PTs. Since the voice biomarker itself had an AUC of 0.79, only when the AUC of gene >0.65 (PT > 0.0005) can the combined biomarker perform better than voice only.

When only PRS was entered in the logistic model, it accounted for 82.9% of the variance in the dependent variable MDD (Nagelkerke’s *R* ^2^). Combined with voice, the Nagelkerke’s *R* ^2^ was 90.2%, indicating that the unique contribution of voice features was 8.7%. Furthermore, we illustrated how genes and voice work together to improve the predictive power by stratifying the test sample according to genetic risks and calculating the accuracies by stratifications. The results of the genes in identifying MDD for both high- and low-genetic-risk populations were consistent with the high accuracy (0.90). However, for the middle population, the accuracy of genes was poor (0.64), due mainly to the inability of genetic features to measure the effect of MDD-related environmental factors. Meanwhile, the accuracy of voice was consistent across the different genetic risk populations, suggesting that the predictive ability of voice was independent of genetic characteristics and that voice capture information was independent of genes. As a result, combining gene and voice biomarkers can effectively improve the detection ability of MDD.

We further explored whether different ML models can further improve the prediction of MDD. For the ML models, we tested the results using SVM, RF, and MLP and compared them with the results of LR. The results (Table 2 and Supplementary Data Sheet S3) showed that MLP could indeed further improve the prediction of the model, improving the AUC by 2.5% with PT = 0.001 and by 3.6% with PT = 0.005.

There are several limitations in this research. To ensure homogeneity between subjects, this study selected women with recurrent MDD as cases, and 85% of the cases met the DSM-IV criteria for melancholia, which is a severe subtype of MDD (CONVERGE Consortium 2015). Thus, our samples represent the two poles of the distribution of depression severity in natural populations. Although our experiments effectively demonstrated that the combination of genes and voice could further improve their ability to identify MDD, experimental results based on a more general population are needed before clinical application.

## 5 Conclusion

This study combines the PRS and voice i-vectors to evaluate their ability to detect MDD. PRSs are calculated at different PTs. With the *p*-value threshold at 0.005, the combined biomarker improved the AUC by 9.09% compared to genes only and 6.73% compared to voice only. Genetic risk stratification analysis showed that the ability for MDD detection of voice is genetically independent. Multilayer perceptron further improved the AUC by 3.6% compared to logistic regression. The combination of PRS and voice biomarkers in MDD detection is feasible. This study provides a foundation for exploring the clinical application of genetic and voice biomarkers in the diagnosis of MDD (Wray et al., 2018).

## Data Availability

Publicly available datasets were analyzed in this study. The data can be found here: https://www.ebi.ac.uk/ena/browser/view/PRJNA289433?show=related-records.

## References

[B1] Alexopoulos G. S. Meyers B. S. Young R. C. Campbell S. Silbersweig D. Charlson M. (1997). 'Vascular Depression' Hypothesis. Arch. Gen. Psychiatry 54 (10), 915–922. 10.1001/archpsyc.1997.01830220033006 PubMed Abstract | 10.1001/archpsyc.1997.01830220033006 | Google Scholar 9337771

[B2] Association A. P. (1994). Diagnostic and Statistical Manual of Mental Disorders. Washington, D.C: American Psychiatric Association. Google Scholar

[B3] Badhwar A. McFall G. P. Sapkota S. Black S. E. Chertkow H. Duchesne S. (2020). A Multiomics Approach to Heterogeneity in Alzheimer's Disease: Focused Review and Roadmap. Brain 143 (May), 1315–1331. 10.1093/brain/awz384 PubMed Abstract | 10.1093/brain/awz384 | Google Scholar 31891371 PMC7241959

[B5] Chang C. C. Chow C. C. Tellier L. C. Vattikuti S. Purcell S. M. Lee J. J. (2015). Second-Generation PLINK: Rising to the Challenge of Larger and Richer Datasets. GigaSci 4 (1), 7. 10.1186/s13742-015-0047-8 PubMed Abstract | 10.1186/s13742-015-0047-8 | Google Scholar PMC4342193 25722852

[B6] Chatterjee N. Shi J. García-Closas M. (2016). Developing and Evaluating Polygenic Risk Prediction Models for Stratified Disease Prevention. Nat. Rev. Genet. 17 (7), 392–406. 10.1038/nrg.2016.27 PubMed Abstract | 10.1038/nrg.2016.27 | Google Scholar 27140283 PMC6021129

[B8] CONVERGE consortium (2015). Sparse Whole-Genome Sequencing Identifies Two Loci for Major Depressive Disorder. Nature 523 (7562), 588–591. 10.1038/nature14659 PubMed Abstract | 10.1038/nature14659 | Google Scholar 26176920 PMC4522619

[B9] Cummins N. Epps J. Sethu V. Krajewski J. (2014).Variability Compensation in Small Data: Oversampled Extraction of I-Vectors for the Classification of Depressed Speech, Proceeding of the 2014 IEEE International Conference on Acoustics, Speech and Signal Processing (ICASSP), May 2014, Florence, Italy. IEEE, 970–974. 10.1109/ICASSP.2014.6853741 10.1109/ICASSP.2014.6853741 | Google Scholar

[B10] Dehak N. Kenny P. J. Dehak R. Dumouchel P. Ouellet P. (2011). Front-End Factor Analysis for Speaker Verification. IEEE Trans. Audio Speech Lang. Process. 19 (4), 788–798. 10.1109/tasl.2010.2064307 10.1109/tasl.2010.2064307 | Google Scholar

[B11] Demyttenaere K. Bruffaerts R. Posada-Villa J. Gasquet I. Kovess V. Lepine J. P. (2004). Prevalence, Severity, and Unmet Need for Treatment of Mental Disorders in the World Health Organization World Mental Health Surveys. Jama 291 (21), 2581–2590. 10.1001/jama.291.21.2581 PubMed Abstract | 10.1001/jama.291.21.2581 | Google Scholar 15173149

[B12] Di Y. Wang J. Li W. Zhu T. (2021). Using I-Vectors from Voice Features to Identify Major Depressive Disorder. J. Affective Disord. 288 (June), 161–166. 10.1016/j.jad.2021.04.004 10.1016/j.jad.2021.04.004 | Google Scholar PMC11681263 33895418

[B13] Euesden J. Lewis C. M. O’Reilly P. F. (2015). PRSice: Polygenic Risk Score Software. Bioinformatics 31 (9), 1466–1468. 10.1093/bioinformatics/btu848 PubMed Abstract | 10.1093/bioinformatics/btu848 | Google Scholar 25550326 PMC4410663

[B15] Goldberg D. (1995). Epidemiology of Mental Disorders in Primary Care Settings. Epidemiologic Rev. 17 (1), 182–190. 10.1093/oxfordjournals.epirev.a036174 PubMed Abstract | 10.1093/oxfordjournals.epirev.a036174 | Google Scholar 8521936

[B16] Gustafsson H. Nordstrom A. Nordstrom P. (2015). Depression and Subsequent Risk of Parkinson Disease: A Nationwide Cohort Study. Neurology 84 (24), 2422–2429. 10.1212/wnl.0000000000001684 PubMed Abstract | 10.1212/wnl.0000000000001684 | Google Scholar 25995056 PMC4478031

[B17] Hoang T. Nguyen Ngoc Q. Lee J. Lee E. K. Hwangbo Y. Kim J. (2021). Evaluation of Modifiable Factors and Polygenic Risk Score in Thyroid Cancer. Endocrine-Related Cancer 28 (7), 481–494. 10.1530/ERC-21-0078 PubMed Abstract | 10.1530/ERC-21-0078 | Google Scholar 33999009

[B18] Howard D. M. Adams M. J. Adams M. J. Clarke T.-K. Hafferty J. D. Gibson J. (2019). Genome-Wide Meta-Analysis of Depression Identifies 102 Independent Variants and Highlights the Importance of the Prefrontal Brain Regions. Nat. Neurosci. 22 (3), 343–352. 10.1038/s41593-018-0326-7 PubMed Abstract | 10.1038/s41593-018-0326-7 | Google Scholar 30718901 PMC6522363

[B19] Kapoor P. M. Middha P. Mavaddat N. Choudhury P. P. Wilcox A. N. Lindstrom S. (2021). Combined Associations of a Polygenic Risk Score and Classical Risk Factors with Breast Cancer Risk. Jnci-Journal Natl. Cancer Inst. 113 (3), 329–337. 10.1093/jnci/djaa056 10.1093/jnci/djaa056 | Google Scholar PMC7936056 32359158

[B20] Kendler K. S. Aggen S. H. Neale M. C. (2013). Evidence for Multiple Genetic Factors Underlying DSM-IV Criteria for Major Depression. JAMA Psychiatry 70 (6), 599–607. 10.1001/jamapsychiatry.2013.751 PubMed Abstract | 10.1001/jamapsychiatry.2013.751 | Google Scholar 23740048 PMC3800168

[B21] Kendler K. S. Gardner C. O. Neale M. C. Prescott C. A. (2001). Genetic Risk Factors for Major Depression in Men and Women: Similar or Different Heritabilities and Same or Partly Distinct Genes. Psychol. Med. 31 (4), 605–616. 10.1017/s0033291701003907 PubMed Abstract | 10.1017/s0033291701003907 | Google Scholar 11352363

[B22] Kendler K. S. Gatz M. Gardner C. O. Pedersen N. L. (2006). A Swedish National Twin Study of Lifetime Major Depression. Am. J. Psychiatry 163 (1), 109–114. 10.1176/appi.ajp.163.1.109 10.1176/appi.ajp.163.1.109 | Google Scholar 16390897

[B23] Kendler K. S. Gatz M. Gardner C. O. Pedersen N. L. (2007). Clinical Indices of Familial Depression in the Swedish Twin Registry. Acta Psychiatr. Scand. 115 (3), 214–220. 10.1111/j.1600-0447.2006.00863.x PubMed Abstract | 10.1111/j.1600-0447.2006.00863.x | Google Scholar 17302621

[B24] Kenny P. Boulianne G. Dumouchel P. (2005). Eigenvoice Modeling with Sparse Training Data. IEEE Trans. Speech Audio Process. 13 (3), 345–354. 10.1109/TSA.2004.840940 10.1109/TSA.2004.840940 | Google Scholar

[B25] Kenny P. Ouellet P. Dehak N. Gupta V. Dumouchel P. (2008). A Study of Interspeaker Variability in Speaker Verification. IEEE Trans. Audio Speech Lang. Process. 16 (5), 980–988. 10.1109/TASL.2008.925147 10.1109/TASL.2008.925147 | Google Scholar

[B26] Kessler R. C. Berglund P. Demler O. Jin R. Koretz D. Merikangas K. R. (2003). The Epidemiology of Major Depressive Disorder. JAMA 289 (23), 3095–3105. 10.1001/jama.289.23.3095 PubMed Abstract | 10.1001/jama.289.23.3095 | Google Scholar 12813115

[B27] Li G. Zhu H. (2013). Genetic Studies: The Linear Mixed Models in Genome-wide Association Studies. Open Bioinformatics J. 7 (1), 27–33. 10.2174/1875036201307010027 10.2174/1875036201307010027 | Google Scholar

[B28] Lippert C. Listgarten J. Liu Y. Kadie C. M. Davidson R. I. Heckerman D. (2011). FaST Linear Mixed Models for Genome-wide Association Studies. Nat. Methods 8 (10), 833–835. 10.1038/nmeth.1681 PubMed Abstract | 10.1038/nmeth.1681 | Google Scholar 21892150

[B29] Low D. M. Bentley K. H. Ghosh S. S. (2020). Automated Assessment of Psychiatric Disorders Using Speech: A Systematic Review. Laryngoscope Invest. Otolaryngol. 5 (1), 96–116. 10.1002/lio2.354 10.1002/lio2.354 | Google Scholar PMC7042657 32128436

[B30] Masters M. C. Morris J. C. Roe C. M. (2015). "Noncognitive" Symptoms of Early Alzheimer Disease: A Longitudinal Analysis. Neurology 84 (6), 617–622. 10.1212/wnl.0000000000001238 PubMed Abstract | 10.1212/wnl.0000000000001238 | Google Scholar 25589671 PMC4335988

[B31] Moldovan A. Waldman Y. Y. Brandes N. Linial M. (2021). Body Mass Index and Birth Weight Improve Polygenic Risk Score for Type 2 Diabetes. J. Personalized Med. 11 (6), 582. 10.3390/jpm11060582 10.3390/jpm11060582 | Google Scholar PMC8233887 34205563

[B32] Mullins N. Bigdeli T. B. Børglum A. D. Coleman J. R. I. Demontis D. Mehta D. (2019). GWAS of Suicide Attempt in Psychiatric Disorders and Association with Major Depression Polygenic Risk Scores. Am. J. Psychiatry 176 (8), 651–660. 10.1176/appi.ajp.2019.18080957 PubMed Abstract | 10.1176/appi.ajp.2019.18080957 | Google Scholar 31164008 PMC6675659

[B33] Murray G. K. Lin T. Austin J. McGrath J. J. Hickie I. B. Wray N. R. (2021). Could Polygenic Risk Scores Be Useful in Psychiatry. JAMA Psychiatry 78 (2), 210. 10.1001/jamapsychiatry.2020.3042 PubMed Abstract | 10.1001/jamapsychiatry.2020.3042 | Google Scholar 33052393

[B34] Nagelkerke N. J. D. (1991). A Note on a General Definition of the Coefficient of Determination. Biometrika 78 (3), 691–692. 10.1093/biomet/78.3.691 10.1093/biomet/78.3.691 | Google Scholar

[B35] Pedregosa F. Varoquaux G. Gramfort A. Michel V. Thirion B. Grisel O. (2011). Scikit-Learn: Machine Learning in Python. J. Machine Learn. Res. 12, 2825–2830. 10.5555/1953048.2078195 10.5555/1953048.2078195 | Google Scholar

[B36] Peterson R. E. Cai N. Dahl A. W. Bigdeli T. B. Edwards A. C. Webb B. T. (2018). Molecular Genetic Analysis Subdivided by Adversity Exposure Suggests Etiologic Heterogeneity in Major Depression. Am. J. Psychiatry 175 (6), 545. 10.1176/appi.ajp.2017.17060621 PubMed Abstract | 10.1176/appi.ajp.2017.17060621 | Google Scholar 29495898 PMC5988935

[B37] Povey D. Ghoshal A. Boulianne G. Burget L. Glembek O. Goel N. (2011).The Kaldi Speech Recognition Toolkit, IEEE 2011 Workshop on Automatic Speech Recognition and Understanding. IEEE Signal Processing Society. Google Scholar

[B38] Rantalainen V. Binder E. B. Lahti‐Pulkkinen M. Czamara D. Laivuori H. Villa P. M. (2020). Polygenic Prediction of the Risk of Perinatal Depressive Symptoms. Depress. Anxiety 37 (9), 862–875. 10.1002/da.23066 PubMed Abstract | 10.1002/da.23066 | Google Scholar 32627298

[B39] Regier D. A. Narrow W. E. Clarke D. E. Kraemer H. C. Kuramoto S. J. Kuhl E. A. (2013). DSM-5 Field Trials in the United States and Canada, Part II: Test-Retest Reliability of Selected Categorical Diagnoses. Am. J. Psychiatry 170 (1), 59–70. 10.1176/appi.ajp.2012.12070999 10.1176/appi.ajp.2012.12070999 | Google Scholar 23111466

[B40] Reus L. M. Shen X. Gibson J. Wigmore E. Ligthart L. Adams M. J. (2017). Association of Polygenic Risk for Major Psychiatric Illness with Subcortical Volumes and White Matter Integrity in UK Biobank. Sci. Rep. 7 (1), 42140. 10.1038/srep42140 PubMed Abstract | 10.1038/srep42140 | Google Scholar 28186152 PMC5301496

[B41] the Major Depressive Disorder Working Group of the Psychiatric Genomics Consortium Ripke S. Mattheisen M. Trzaskowski M. Byrne E. M. Abdellaoui A. Adams M. J. (2018). Genome-Wide Association Analyses Identify 44 Risk Variants and Refine the Genetic Architecture of Major Depression. Nat. Genet. 50 (5), 668–681. 10.1038/s41588-018-0090-3 PubMed Abstract | 10.1038/s41588-018-0090-3 | Google Scholar 29700475 PMC5934326

[B42] Schneider B. Prvulovic D. (2013). Novel Biomarkers in Major Depression. Curr. Opin. Psychiatry 26 (1), 47–53. 10.1097/YCO.0b013e32835a5947 PubMed Abstract | 10.1097/YCO.0b013e32835a5947 | Google Scholar 23154643

[B43] Shin D. Cho W. I. Park C. H. K. Rhee S. J. Kim M. J. Lee H. (2021). Detection of Minor and Major Depression through Voice as a Biomarker Using Machine Learning. J. Clin. Med. 10 (14), 3046. 10.3390/jcm10143046 10.3390/jcm10143046 | Google Scholar 34300212 PMC8303477

[B44] Sullivan P. F. Neale M. C. Kendler K. S. (2000). Genetic Epidemiology of Major Depression: Review and Meta-Analysis. Am. J. Psychiatry 157 (10), 1552–1562. 10.1176/appi.ajp.157.10.1552 10.1176/appi.ajp.157.10.1552 | Google Scholar 11007705

[B45] Torkamani A. Wineinger N. E. Topol E. J. (2018). The Personal and Clinical Utility of Polygenic Risk Scores. Nat. Rev. Genet. 19 (9), 581–590. 10.1038/s41576-018-0018-x PubMed Abstract | 10.1038/s41576-018-0018-x | Google Scholar 29789686

[B46] Wang J. Zhang L. Liu T. Pan W. Hu B. Zhu T. (2019). Acoustic Differences between Healthy and Depressed People: A Cross-Situation Study. BMC Psychiatry 19 (1), 300. 10.1186/s12888-019-2300-7 PubMed Abstract | 10.1186/s12888-019-2300-7 | Google Scholar 31615470 PMC6794822

[B47] Wells K. B. Hays R. D. Burnam M. A. Rogers W. Greenfield S. Ware J. E. (1989). Detection of Depressive Disorder for Patients Receiving Prepaid or Fee-For-Service Care. Jama 262 (23), 3298–3302. 10.1001/jama.1989.03430230083030 PubMed Abstract | 10.1001/jama.1989.03430230083030 | Google Scholar 2585674

[B48] World Health Organization (2017). Depression and Other Common Mental Disorders: Global Health Estimates. Available at: https://www.who.int/publications/i/item/depression-global-health-estimates (Accessed December 6, 2021). Google Scholar

[B49] Wray N. R. Goddard M. E. Visscher P. M. (2007). Prediction of Individual Genetic Risk to Disease from Genome-wide Association Studies. Genome Res. 17 (10), 1520–1528. 10.1101/gr.6665407 PubMed Abstract | 10.1101/gr.6665407 | Google Scholar 17785532 PMC1987352

[B50] Zhang L. Duvvuri R. Chandra K. K. L. Nguyen T. Ghomi R. H. Ghomi R. H. (2020). Automated Voice Biomarkers for Depression Symptoms Using an Online Cross‐sectional Data Collection Initiative. Depress. Anxiety 37 (7), 657–669. 10.1002/da.23020 PubMed Abstract | 10.1002/da.23020 | Google Scholar 32383335

