# Supplementary material for: Combining Polygenic Risk Score and Voice Features to Detect Major Depressive Disorders
Source: Front Genet. 2021 Dec 20;12:761141. doi: 10.3389/fgene.2021.761141 (PMC8721147; doi:10.3389/fgene.2021.761141)
Supplement: Supplementary file 1 [file DataSheet3.docx]

**Supplementary**

Table S1 AUC with different PT using different ML models.

| PT | LR | SVM | RF | MLP |
| --- | --- | --- | --- | --- |
| 5E-08 | 0.76 | 0.75 | 0.70 | 0.77 |
| 1E-06 | 0.76 | 0.76 | 0.70 | 0.77 |
| 1E-05 | 0.76 | 0.76 | 0.70 | 0.77 |
| 5E-05 | 0.76 | 0.76 | 0.70 | 0.77 |
| 1E-04 | 0.76 | 0.76 | 0.70 | 0.78 |
| 5E-04 | 0.78 | 0.78 | 0.72 | 0.80 |
| 0.001 | 0.79 | 0.79 | 0.74 | 0.81 |
| 0.005 | 0.83 | 0.83 | 0.80 | 0.86 |

Table S2 Sensitivity with different PT using different ML models.

| PT | LR | SVM | RF | MLP |
| --- | --- | --- | --- | --- |
| 5E-08 | 0.75 | 0.75 | 0.68 | 0.80 |
| 1E-06 | 0.75 | 0.76 | 0.68 | 0.79 |
| 1E-05 | 0.75 | 0.76 | 0.68 | 0.79 |
| 4E-05 | 0.76 | 0.76 | 0.68 | 0.79 |
| 5E-05 | 0.76 | 0.75 | 0.67 | 0.80 |
| 1E-04 | 0.76 | 0.75 | 0.67 | 0.81 |
| 5E-04 | 0.78 | 0.78 | 0.68 | 0.81 |
| 0.001 | 0.79 | 0.80 | 0.70 | 0.83 |
| 0.005 | 0.83 | 0.83 | 0.78 | 0.87 |

Table S3 Specificity with different PT using different ML models.

| PT | LR | SVM | RF | MLP |
| --- | --- | --- | --- | --- |
| 5E-08 | 0.76 | 0.75 | 0.72 | 0.75 |
| 1E-06 | 0.76 | 0.76 | 0.72 | 0.74 |
| 1E-05 | 0.76 | 0.76 | 0.73 | 0.75 |
| 4E-05 | 0.76 | 0.76 | 0.73 | 0.75 |
| 5E-05 | 0.76 | 0.76 | 0.73 | 0.75 |
| 1E-04 | 0.76 | 0.76 | 0.74 | 0.76 |
| 5E-04 | 0.78 | 0.78 | 0.76 | 0.78 |
| 0.001 | 0.78 | 0.78 | 0.77 | 0.79 |
| 0.005 | 0.83 | 0.83 | 0.81 | 0.85 |
